# Optomechanical switching of adsorption configurations of polar organic molecules by UV radiation pressure

**DOI:** 10.1038/s41598-021-92046-w

**Published:** 2021-06-16

**Authors:** Kowsalya Arumugam, Abhishake Goyal, Hong-Ming Chen, Jing-Huan Dai, Mau-Fu Gao, Yasuo Nakayama, Tun-Wen Pi, Theodoros A. Papadopoulos, Horng-Tay Jeng, Shu-Jung Tang

**Affiliations:** 1 Department of Physics and Astronomy, National Tsing Hua University, Hsinchu, 30013 Taiwan, ROC; 2 Department of Pure and Applied Chemistry, Tokyo University of Science, 2641 Yamazaki, Noda, Chiba 278-8510 Japan; 3 National Synchrotron Radiation Research Center (NSRRC), Hsinchu, 30076 Taiwan, ROC; 4 Department of Mathematical and Physical Sciences, University of Chester, Thornton Science Park, Chester, CH2 4NU UK; 5 Physics Division, National Center for Theoretical Sciences, Hsinchu, 30013 Taiwan, ROC; 6 Institute of Physics, Academia Sinica, Taipei, 11529 Taiwan, ROC

**Keywords:** Chemical physics, Physics, Condensed-matter physics, Surfaces, interfaces and thin films

## Abstract

Using photoemission spectroscopy (PES), we have systematically investigated the behavior of polar organic molecule, chloroaluminum phthalocyanine (ClAlPc), adsorbed in the Cl-down configuration on the Ag(111) substrate at low temperature − 195 °C under UV irradiation with a range of different photon fluxes. Judging from the evolution of photoemission spectral line shapes of molecular energy states, we discovered that the Cl atoms are so robustly anchored at Ag(111) that the impinging photons cannot flip the ClAlPc molecules, but instead they crouch them down due to radiation pressure; we observe that the phthalocyanine (Pc) lobes bend down to interact with Ag atoms on the substrate and induce charge transfer from them. As photon flux is increased, radiation pressure on the Pc plane initiates tunneling of the Cl atom through the molecular plane to turn the adsorption configuration of ClAlPc from Cl-down to an upheld Cl-up configuration, elucidating an optomechanical way of manipulating the dipole direction of polar molecules. Finally, work function measurements provide a distinct signature of the resulting upheld Cl-up configuration as it leads to a large increase in vacuum level (VL), ~ 0.4 eV higher than that of a typical flat-on Cl-up configuration driven by thermal annealing.

## Introduction

Using light to induce mechanical motion of a molecule has been a hot research topic^1^ since light is easily controlled in the intensity, frequency, and spot size with instant reaction time from molecules and compatibility in ambient environments. Moreover, molecular machines or devices powered by lights are perspective devices in nanoscales for industrial applications^2–4^. A major way that has been widely employed is the photoisomerization effect that allows a molecule to switch between *cis* and *trans* states because of the change in bond angle (*cis* is shorter and *trans* is extended) leading to the alternation of geometry and function of molecular complexes^5–7^. Such a way has been applied to various light-driven macroscopic motions, e.g., the deformation of a liquid droplet due to the change of surface free energy of the underlying molecular monolayer, and optomechanical cycle of individual polymers with two ends coupled to the microscopy tip and substrate surface, respectively^5,8,9^. The mechanism of photoisomerization is related to the $n \to \pi^{*}$ and $\pi \to \pi^{*}$ quantum excitations, triggered by lights of different frequencies^5,10,11^. However, a much simpler mechanism, i.e. the effect of radiation pressure inflicted by photon irradiation, has been overlooked. Little is known about how radiation pressure affects molecules adsorbed on a substrate surface. Kowsalya et. *al.* investigated the irradiation effect on the ClAlPc polar organic molecules adsorbed on Ag(111) in the Cl-down configuration at the room temperature (RT) by the PES. They found that ClAlPc molecules tended to be tilted and partially flipped under irradiation, leading to an overall disordered phase as indicated by the broadened spectral line shape of molecular energy states^12^.

Polar organic molecules consisting of a dipole and phthalocyanine (Pc) plane, such as ClAlPc, and its derivatives possess diverse electronic applications like molecular switches^13^**,** chemical sensors^14^, organic diodes^15^, organic photovoltaic logical circuits^16^, and data storage devices^17^. The physical and chemical properties of a monolayer (ML) of polar molecules adsorbed on substrate surfaces depend decisively on the molecular dipole orientation that leads to disparate electronic structures, charge transfer, and energy level alignment at the interface^18^. Therefore, it is very important to develop a method easily manipulating the adsorption configuration, i.e. dipole-up or dipole-down, of polar organic molecules. It has been demonstrated that a ClAlPc molecule on the highly ordered pyrolytic graphite (HOPG) surface can be switched between Cl-down and Cl-up configurations via applying a positive or negative bias from a tip of scanning tunneling microscopy (STM)^19^ and the potential application to the molecular memory digits was implied^13^. In light of the addressability and practical aspects of light, it is extremely interesting to investigate whether molecular dipole switching can be achieved via radiation. In the experiment presented in this paper, we intentionally froze ClAlPc molecules anchored on Ag(111) in Cl-down configuration by cooling them down to − 195 °C. Then we performed a real-time monitoring of irradiation effects on the molecules by investigating the time-dependent evolution of photoemission spectral line shape of molecular energy states. Strikingly, a ClAlPc crouching motion reinforced by radiation pressure was observed, instigating the Cl atom to tunnel through the Pc plane to cause the complete switching from Cl-down to Cl-up configuration.

## Experimental section

### Sample preparation and work function measurement

PES measurements were performed at the end station 08A1-LSGM in National Synchrotron Radiation Research Center (NSRRC), Taiwan with R3000 Scienta energy analyzer at the photon energy of 50 eV. The photon fluxes were controlled by the slits and measured by the mesh currents. The previously calibrated relation between the photon flux and the mesh currents were used to calculate the photon flux values^12^. The total energy resolution was 50 meV. The work-function measurement was carried out by examining the cutoff energy of secondary electrons with the sample electrically biased at 6.0 V^20^. The ultrahigh vacuum system consists of interconnected sample-preparation (base pressure: 1 × 10^−8^ mbar) and measurement chamber (base pressure: 9.8 × 10^−10^ mbar). The Ag(111) crystal is chosen as the substrate for the organic molecule ClAlPc. The standard sputtering and annealing procedure was used to clean the sample^21^. The clean Ag(111) surface was confirmed by the LEED patterns and the sharp Ag Shockley surface state in photoemission spectra. Then highly purified ClAlPc molecules were evaporated from water-cooled Kundsen-type thermal evaporator to Ag(111) at RT. The deposition rate of ClAlPc molecule was determined by pre-calibrated quartz thickness monitor. Following the procedure previously described^20^, 1-ML ClAlPc in the complete Cl-down configuration and the mixed phase of Cl-up and Cl-down configurations were prepared by lower deposition rate, 0.04 Å/min, and higher deposition rate, 3 Å/min, respectively. The complete Cl-up configuration is formed with further post annealing of the mixed phase to 60 °C^12,20^. After deposition, the sample was then transferred to the measurement chamber and cooled down to − 195 °C using liquid nitrogen to perform the PES measurement. Before each time-dependent photoemission measurement, the before-irradiation spectra was taken at photon flux 2.11 × 10^13^ photons/sec for 5 min to minimize the effect of radiation pressure. During the time-dependent photoemission measurement, 9 sweeps of spectrums were taken (5 min for one sweep) at different photon fluxes (3.14 × 10^13^ photons/sec, 4.18 × 10^13^ photons,sec, and 5.22 × 10^13^ photons/sec). The spectrum from the 9th sweep is defined as after-irradiation spectra.

### Computational methodology

The ab-initio calculations were performed using the Vienna Ab-initio Simulation Package (VASP)^22–25^ based on Density Functional Theory (DFT). The Perdew–Burke-Ernzerhof (PBE)^25^ generalized gradient-approximation functional, and the projector augmented wave (PAW)^26,27^ methods are employed in the self-consistent calculations. The plane-wave cutoff energy is set at 400 eV with a 6×6×1 k-mesh over the two-dimensional Brillouin zone of Ag supercell in all calculations. The ClAlPc/Ag(111) system is simulated by putting a ClAlPc molecule on top of the Ag(111) 6 × 6 supercell with the thickness of 3 Ag layers. The Ag(111) 6 × 6 supercell adopted here is approximately the smallest one that can accommodate the ClAlPc molecule without imposing steric effects. To simulate the bended Cl-up ClAlPc molecule, we fix the Cl atom at several heights of 3.5, 4.0, 4.5, 5.0, and 5.5 Å with respect to the surface Ag layer and perform geometrical optimization for all the other atoms until the total energy converges within 10^−4^ eV for each case. In this way, the evolution of the total energy as a function of Cl atom height is explored, and a local minimum of total energy is discovered with the Cl atom located at height of ~ 4.5 Å.

## Results and discussion

### Absorption configurations of polar ClAlPc molecule

**Figure 1:**
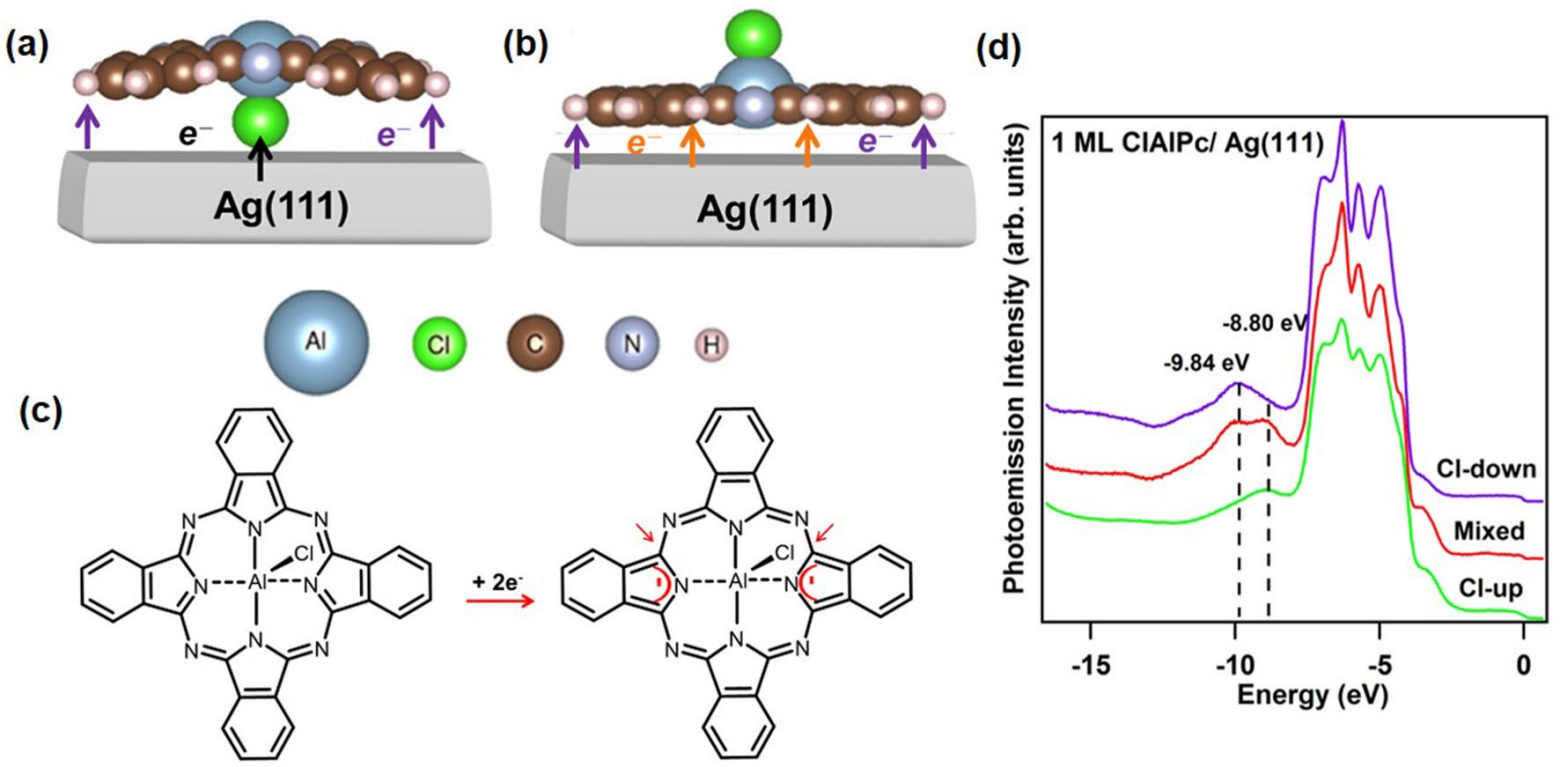
Side views of a ClAlPc molecule on Ag(111) crystal in (**a**) Cl-down configuration and (**b**) Cl-up configuration. The dark, purple, and orange arrows indicate the different charge-transfer channels from Ag(111) to the molecule. (**c**) Chemical structures of neutral ClAlPc and dianionic ClAlPc^2-^^8,11,22,29^. (**d**) Photoemission spectra of 1-ML ClAlPc on Ag(111) crystal surface for Cl-up, mixed, and Cl-down configurations.

Figure 1a and b display schematically the molecular structures of ClAlPc in the Cl-down and Cl-up configurations, which are adsorbed on Ag(111) surfaces. The Al-Cl dipole with a dipole moment of 3.7 Debye^28^ is coupled to the center of Pc, inducing disparate properties of Cl-down and Cl-up configurations. For both adsorption configurations, charge transfer with Ag(111) substrate occurs via three channels^20^, namely, the centered Al-Cl dipole, inner ligand ring, and outer phenylene groups, as indicated by dark, orange and purple arrows. As previously proposed by Niu et al.^29^, Cl-down ClAlPc undergoes Jahn–Teller distortion that causes the observed symmetry reduction from C4 to C2 due to the two Pc lobes bent down toward the substrate surface^29^. Figure 1c depicts the change of the chemical structure of Cl-down ClAlPc from the neutral state to the charged state following the model they proposed in Ref. 29. Due to the larger electronegativity of the Cl atom as compared to that of the Ag atom (Cl: 3.16, Ag:1.93), Cl-down ClAlPc draws electronic charges, indicated by the dark arrow in Fig. 1a, from Ag atoms via Al-Cl dipole and they further transfer through one pair of Al-N bonds across each other, which have shorter bond lengths than another pair perpendicular to them^29^. Consequently, the two isoindole groups connecting to the pair of Al-N bonds accept the charges and further aromatize the two rings, as shown by the red marks in Fig. 1(c), via reducing adjacent N–C double bonds to single ones, as indicated by the red arrows. This weakens the sustention of the corresponding peripheral isoindole units, causing the Pc lobes to bend down toward the Ag surface atoms. Moreover, the charge transfer, as indicated by the purple arrows in Fig. 1a, from Ag atoms to outer phenylene groups are induced as a result of C π-orbital coupling with Ag orbitals to help stabilizing the Cl-down ClAlPc^29^. In terms of the Cl-up configuration, due to being flat on the surface, the interaction between the Pc plane and Ag(111) surface via the C delocalized π bonds also includes the inner-ligand-ring channel, as indicated by the orange arrows in Fig. 1b. Three different configuration states can be clearly observed in Fig. 1d, which shows energy distribution curves (EDCs) for 1-ML Cl-up, Cl-down, and mixed configurations between the two. The molecular-energy-state peaks at − 9.84 and − 8.8 eV represent the Cl-down and Cl-up configuration, respectively, while the two-peak line shape represents a mixed configuration between the two. It was demonstrated that the photoemission spectra line shapes distinctly characterize the adsorption configurations of ClAlPc on Ag(111) after comparison with the computationally acquired energy distributions of partial density of states (DOS) of the composing elements^20^; the peak at − 8.8 eV is mainly contributed by partial DOS of outer phenylene groups and inner ligand rings via the charge-transfer channel indicated by the purple and the orange arrows in Fig. 1b, and the one at − 9.84 eV is mainly contributed by that of the Cl atom via the channel indicated by the dark arrow in Fig. 1a.

### Time-dependent photoemission spectra under photon irradiation

**Figure 2:**
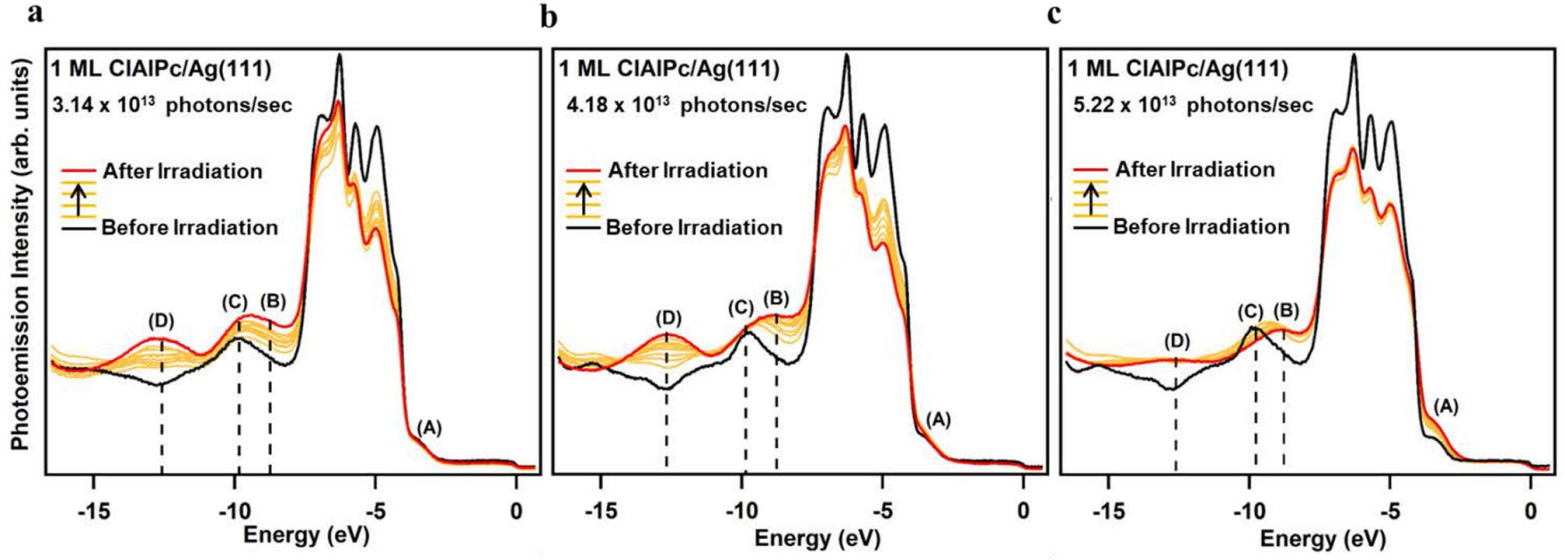
Time-dependent photoemission spectra of 1-ML ClAlPc on Ag(111) at photon flux of (**a**) 3.14 × 10^13^ photons/sec, (**b**) 4.18 × 10^13^ photons/sec, and (**c**) 5.22 × 10^13^ photons/sec. The energy positions of the Cl-down, Cl-up, and anomalous molecular-energy-state peak are indicated with the black dashed line. The consecutive EDCs denoted by the orange color are from the 8 sweeps of the spectrums during irradiation.

Figure 2a–c show the time-dependent EDCs for 1-ML ClAlPc in the Cl-down configuration adsorbed on Ag(111) at − 195 °C under irradiation of a constant photon flux of 3.14 × 10^13^, 4.18 × 10^13^, and 5.22 × 10^13^ photons/sec, respectively. All samples were irradiated for a total time of 45 min each. The five-peak features ranging − 4 ~  − 8 eV were derived from Ag 4*d* states. Before irradiation, the molecular-energy-state peak (peak (C)) representing the Cl-down configuration is observed at the energy position of − 9.84 eV (dark curve). During irradiation, an anomalous peak (peak (D)) emerges at − 12.60 eV and evolves in intensity as time goes by, while the molecular-energy-state peak representing the Cl-down configuration at − 9.84 eV doesn’t just evolve in intensity but also shifts toward the energy position of − 8.8 eV (peak (B)) that is characteristic of the Cl-up configuration. A close examination reveals that irradiation with the largest photon flux, i.e. 5.22 × 10^13^ photons/sec (Fig. 2c), is unique compared to the other two cases employing lower photon fluxes (Fig. 2a,b), in that the anomalous peak at − 12.60 eV undergoes negligible change in intensity and the peak at − 9.84 eV substantially decreases in intensity while shifting in energy towards − 8.8 eV. One can also see the two Ag 4*d-*state peaks at higher energy, − 6 ~  − 4 eV, decay substantially over time for the two cases in Fig. 2a,b, whereas the same two Ag 4*d*-state peaks are pretty robust during irradiation when employing the highest photon flux (Fig. 2c), but a small rising shoulder by the top Ag 4*d*-electron peak is evident at about − 3.5 eV (peak (A)) after irradiation.

**Figure 3:**
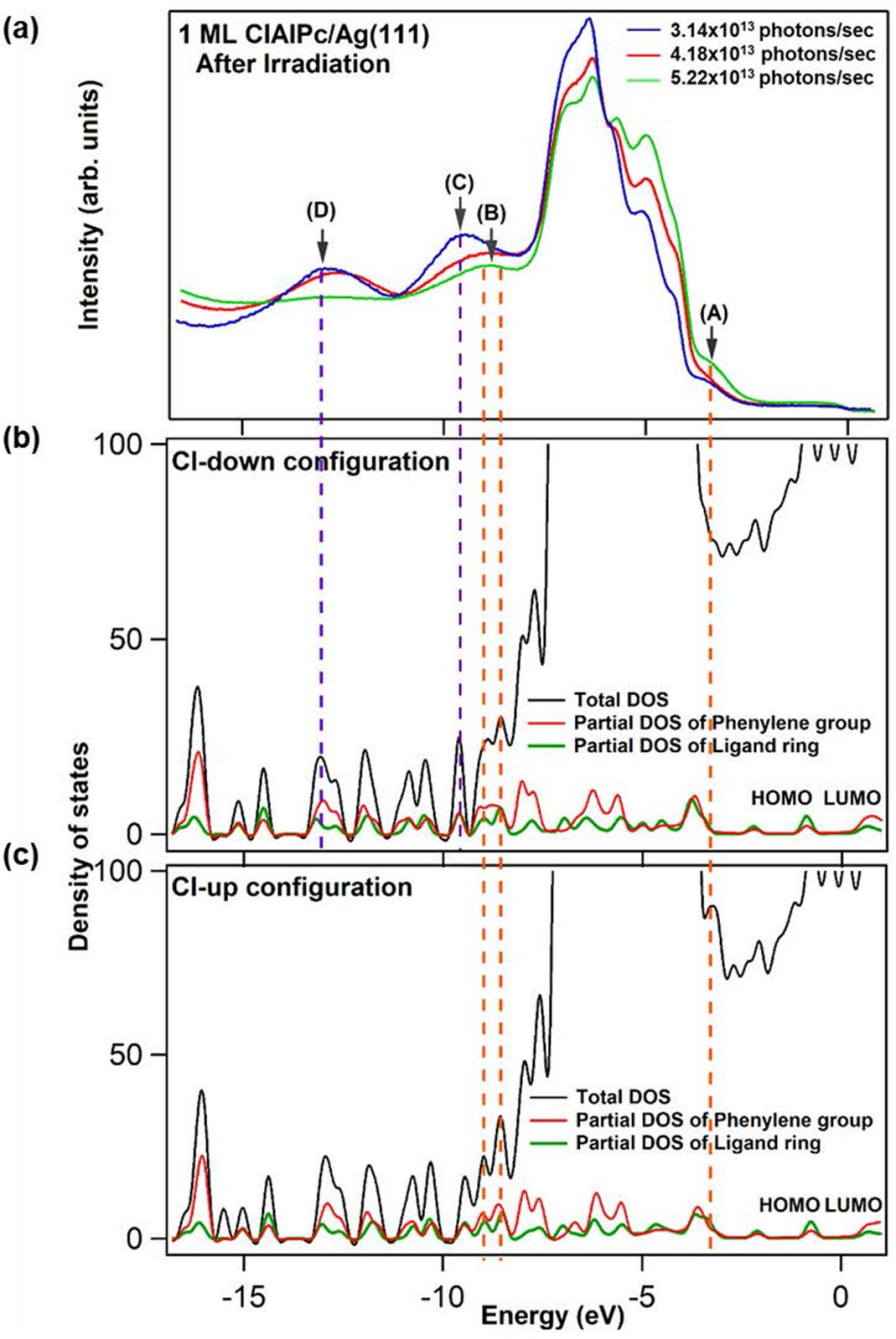
(**a**) After-irradiation photoemission spectra of 1-ML ClAlPc on Ag(111) at the three different photon fluxes. Calculated total and partial DOS for ClAlPc in (**b**) Cl-down configuration and (**c**) Cl-up configuration. The correspondence between the energy positions of molecular-energy-state peaks and those of partial DOS are marked by purple (for Cl-down) and orange (for Cl-up) dashed lines, respectively.

In order to explore the origin of the peak features depicted in Fig. 2, we compare the photoemission data with the total and partial DOS, calculated via DFT in Ref. 20, for the interface between the Ag(111) surface and the Cl-up and Cl-down ClAlPc monolayers in Fig. 3. The final EDC after irradiation for the three initial cases are overlaid in Fig. 3a for clarity and completion. In the energy range − 12 ~  − 14 eV (Fig. 3b and c), the partial DOS contributed from outer phenylene groups is higher than that from inner ligand ring, inferring that the anomalous peak centered at − 12.60 eV, marked by (D), is mainly due to the outer phenylene groups at the Pc periphery.

The trend is clear that peak (C) representing Cl-down configuration has highest intensity at lowest photon flux, while peak (B) representing Cl-up configuration dominates most at highest photon flux. Since the anomalous peak (D) also has highest intensity at lowest photon flux, it is reasonable to ascribe it to the enhanced charge transfer from Ag surface to Pc lobes as the overlap between the C π-bonds and Ag surface orbitals is increased, further consolidating the bond between the Ag surface and Cl-down configuration. It appears that the incident photon flux inflicts radiation pressure on the Pc plane bending the Pc lobes closer to the Ag(111) surface. Note that the intensity of peak (B) also increases at lowest and median photon fluxes during irradiation. It is because partial DOS of the outer phenylene groups contributes not just peak (D) but peak (B) so both increase in intensity consistently. In the energy range − 6 ~  − 4 eV, the partial DOS of outer phenylene groups is equivalent to that of inner ligand ring. The corresponding Ag *d* electron states within that range are from the two outmost *d* shells, which possesses the orbital characters of *d*_zz_, *d*_xz_, and *d*_yz_. Since delocalized π bonds of outer phenylene groups is out of plane, they have most coupling with Ag *d* electrons with orbital symmetry in *z* direction, as manifested by the large intensity reduction for the *d*-electron peaks in that energy range at lowest photon flux. When irradiated at the highest flux, a complete and coherent switch to Cl-up configuration seems to occur because peak (C) keeps decreasing in intensity and vanishes after irradiation as shown in Figs. 2c and Fig. 3a. The Pc plane of Cl-up configuration flat on the Ag(111) surface renders the charge transfer, due to the overlap between the C π-bonds and Ag surface orbitals, both from inner ligand ring and outer phenylene groups (orange and purple arrows in Fig. 1b). In addition, as proposed by Niu et al*.*^29^, significant charge transfer from the substrate atoms to Cl leads to the intake of charge to LUMO to lift its degeneracy and enhance the π-orbital coupling of bent-down Pc lope with substrate states. Therefore the low saturated intensity of the anomalous peak at − 12.60 eV during irradiation (Fig. 2c) indicates not just partial charge-transfer contribution from outer phenylene groups but the breaking of Cl-Ag charge transfer channel (dark arrow in Fig. 1a). It is noteworthy that the peak (A) at − 3.5 eV, which is apparently higher for the case of the highest photon flux, is partially contributed from the DOS of Cl atom for Cl-up configuration^20^.

Therefore the flux of 5.22 × 10^13^ photons/sec appears as a threshold of switchover activation, ruling out the photoisomerization effect which is induced by lights of certain frequencies (photon energies)^5,8^. The force resulting from irradiation can be expressed as $F = \frac{{(\frac{dE}{{dt}})}}{c} = \frac{{\Phi_{photon} \times hv}}{c}$, where $\frac{dE}{{dt}}$, *Φ*_photon_, *hν*, and *c* are the power of light, photon flux, photon energy, and light speed, respectively. With *Φ*_photon_ = 5.22 × 10^13^ photons/sec, *h*ν = 50 eV, *c* = 3 × 10^8^ m/s, the radiation force is 1.39 × 10^−12^ N. However, this is just the force onto an atom. A ClAlPc molecule has 56 atoms in the Pc plane. With one more atom, Al atom, included, the total radiation force onto the Al-Cl dipole is 79.2 pN. Although the actual force constant of Al-Cl dipole is not known, this magnitude of force falls within the same order of the force to move an atom on a substrate surface according to a previous study^30^.

### Proposed optomechanical model

**Figure 4:**
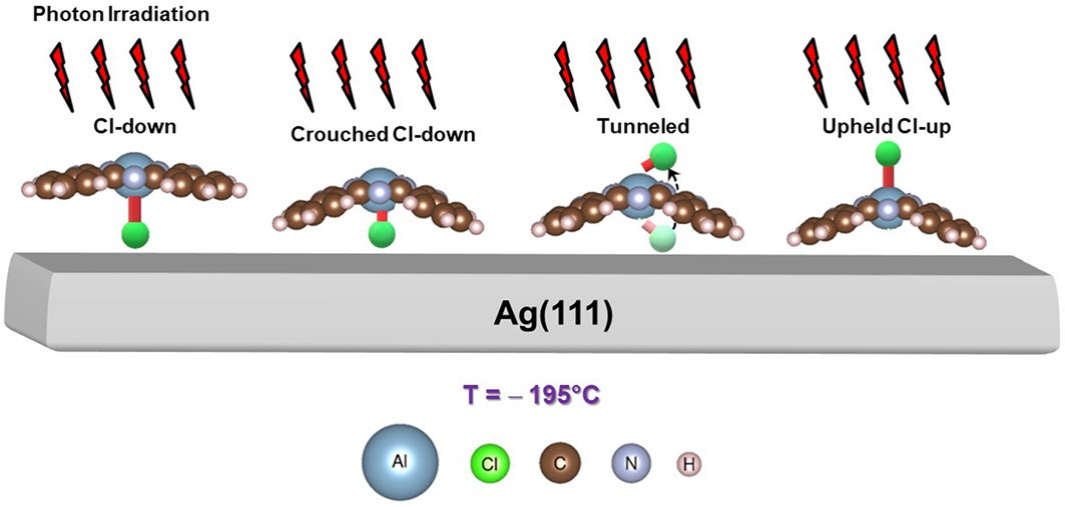
Schematic illustration of the proposed optomechanical model for the tunneling of the Cl atom through the Pc plane of ClAlPc to complete the switchover from Cl-down to upheld Cl-up configuration under photon irradiation.

Figure 4 schematically depicts our optomechanical model proposed in this work for the complete switch of the ClAlPc molecule from the Cl-down to Cl-up configuration. On a typical adsorption condition where the substrate is at RT, the Cl atom of Cl-down ClAlPc draws charges from the Ag substrate and the resulting charge depletion within Al-Cl bond increases the bond length^29^. When the substrate temperature is dropped to − 195 °C, the Al-Cl bond contracts in light of positive thermal expansion due to the anharmonic-potential nature between atoms^31,32^, hence reversely retracting the charge back to the substrate from the Cl atom, weakening the interfacial bonding between Cl and Ag atoms. Radiation pressure on the Pc plane of ClAlPc further compresses the Al-Cl bond to the extent that the Cl-Ag bonding breaks and the Al-Cl dipole then swings with Al as the pivot to tunnel through Pc plane to another side, while the two bent-down Pc lobes further consolidate the anchoring into Ag atoms due to the radiation pressure on Pc plane. This model is completely different from our previous work^12^ (see the Supporting Information), in which the entire process was at RT when ClAlPc underwent irradiation effect; the impinging photons tend to flip the Cl-down ClAlPc and cause only partial switch to the Cl-up configuration because complete flipping requires additional energy. Our present result shows that large reduction in thermal energy can lead the ClAlPc to take the energy-efficient path of tunneling under irradiation to attain the complete configuration switchover.

**Figure 5:**
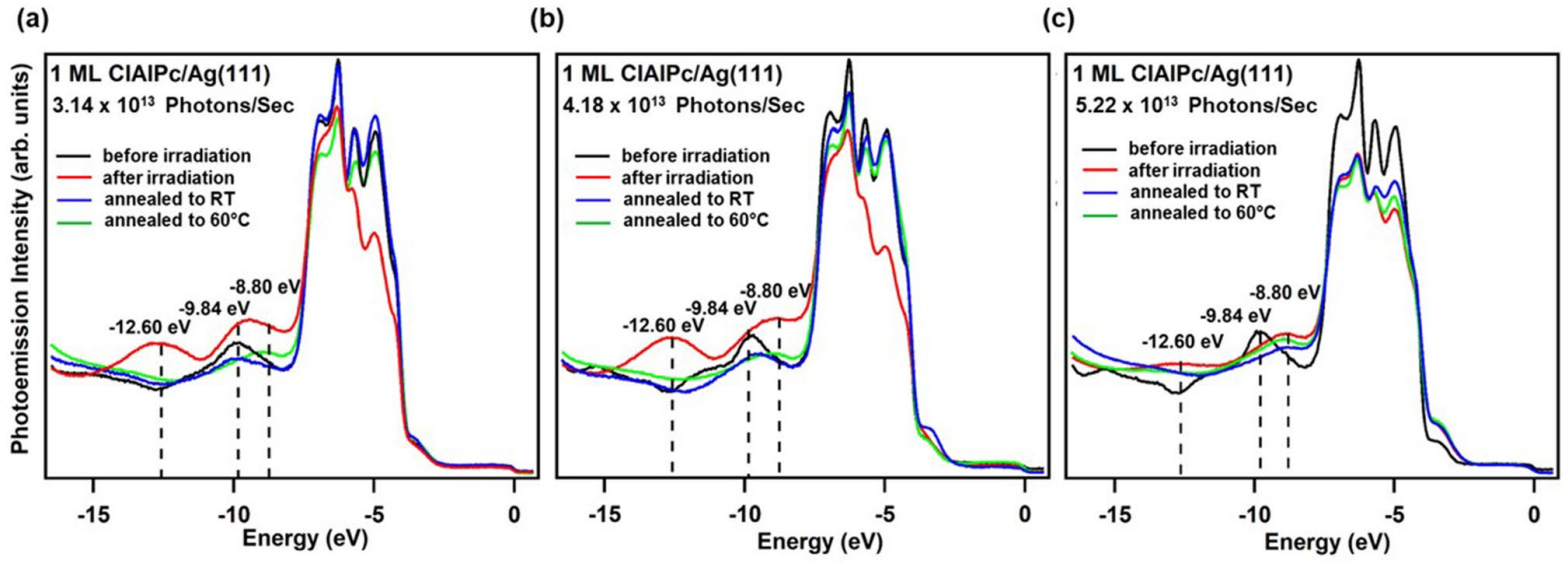
Photoemission spectra of 1-ML ClAlPc on Ag(111) crystal at the stages of before-irradiation, after-irradiation, post-annealed-to-RT, and post-annealed-to-60 °C at photon flux of (**a**) 3.14 × 10^13^ photons/sec, (**b**) 4.18 × 10^13^ photons/sec, and (**c**) 5.22 × 10^13^ photons/sec. The black dashed lines indicate the energy positions of the Cl-down, Cl-up and anomalous molecular-energy-state peaks.

To investigate the effect of ambient-temperature variation in the three cases of Figs. 2 and 3, we further look into the photoemission spectra with the conditions of being further annealed from − 195 °C to RT and 60 °C, respectively. After annealing process, the temperature during photoemission measurement is kept at RT for both. Their EDCs are shown in Fig. 5 together with those before and after irradiation for clarity of comparison. For the case of radiation fluxes of 3.14 × 10^13^ and 4.18 × 10^13^ photons/sec (hereafter denoted as case 1 and case 2), the broad molecular-energy-state peak ranging − 9.84 ~ − 8.80 eV shifts back toward − 9.84 eV upon temperature increase to RT (blue curve), indicating failure to switch from the Cl-down to Cl-up configuration. However for the case of the highest radiation flux (hereafter denoted as case 3), 5.22 × 10^13^ photons/sec, the molecular-energy-state peak stays at about − 8.80 eV after a temperature increase to RT (blue curve), indicating a successful switch. As shown from the green EDCs, further post-annealing to 60 °C (green curve) makes the switch to Cl-up configurations for all three cases as expected^21^.

**Figure 6:**
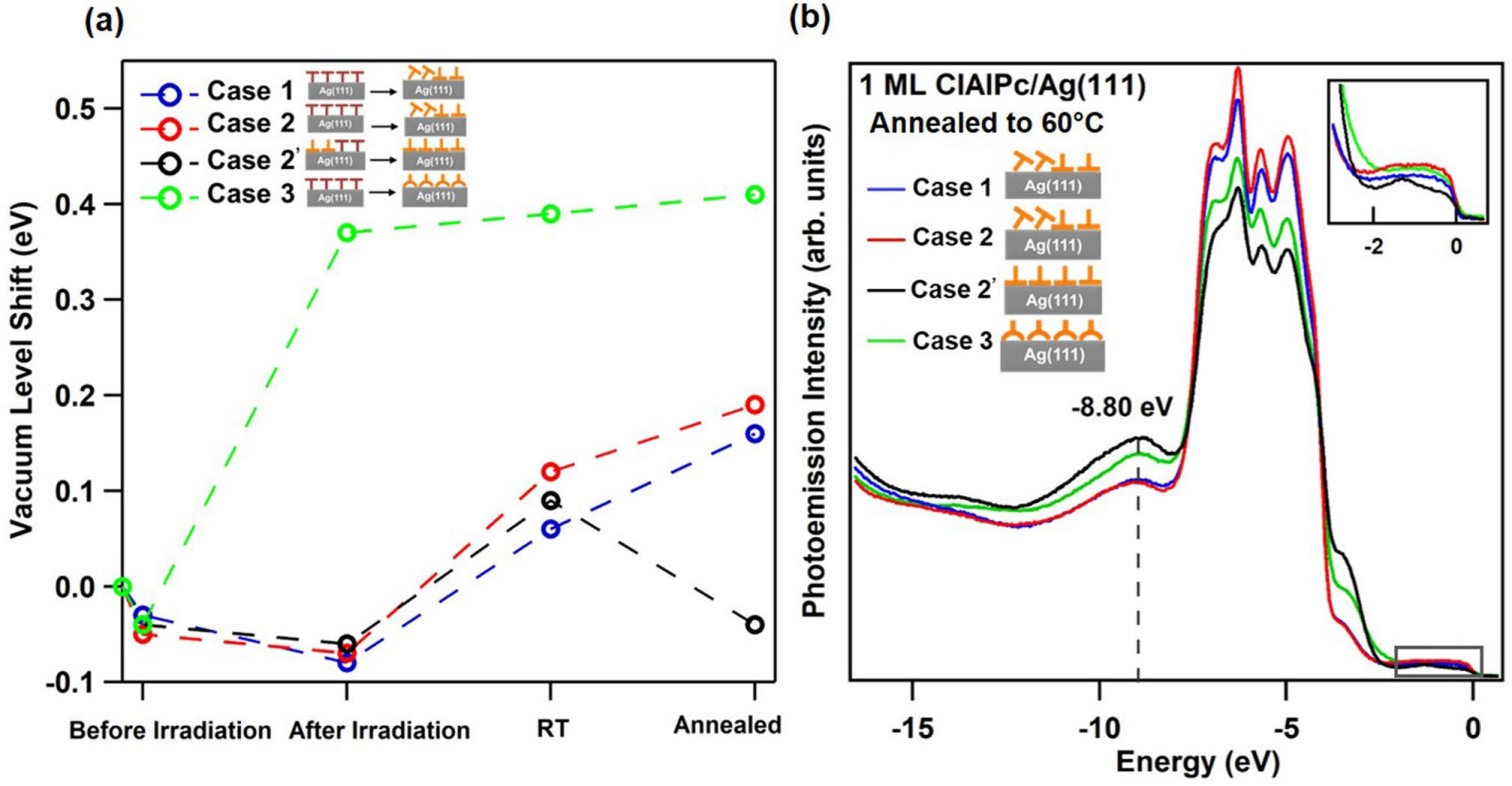
(**a**) VL shifts at the four stages of before-irradiation, after-irradiation, post-annealed-to-RT, and post-annealed-to-60 °C for the four different cases. The zero value of VL shift is with respect to Ag(111). The graphic annotation indicates the transition from the initial stage of before-irradiation to the final stage of post-annealed-to-60 °C. (**b**) Photoemission spectra at the stage of post-annealed-to-60 °C for the four different cases. The black dashed line indicates the energy position of the Cl-up molecular-energy-state peak. The inset magnify the spectra in the energy range near Fermi level.

Note that the final Cl-up configuration derived from the optomechanical model as shown in Fig. 4 is disparate from that shown in Fig. 1b; the Pc plane of the former is still being upheld by the Pc lobes due to their strong anchoring to the substrate while that of the latter is flat on the substrate, which was considered as a typical case^18,20^. To distinguish between these two cases, we investigated the vacuum-level (VL) shifts, as shown in Fig. 6a, on the four stages, namely, before-irradiation, after-irradiation, post-annealed to RT, and post-annealed to 60 °C. Moreover, in addition to the three cases (cases 1–3) discussed in Figs. 2 and 3, we included a case (hereafter denoted as case 2’), in which the initial configuration of ClAlPc before irradiation is a mixture of Cl-up and Cl-down (red curve in Fig. 1d), and the corresponding radiation flux is the same as case 2. Previous studies^12^ show that post annealing to 60 °C from RT causes the initial 1-ML of mixed configurations and 1-ML of the Cl-down configuration on Ag(111) to transit to the complete and incomplete Cl-up configurations, respectively. The Cl-up configuration referred to was the typical flat-on as shown in Fig. 1b. In Fig. 6a, one can see the VL shifts of ClAlPc in the four cases before irradiation are all negative, contrasting positive VL shifts of previous results^12,20^. However, in the present case, the temperature is − 195 °C rather than RT so that the Al-Cl dipole contracts to weaken the interfacial Cl-Ag dipole that mainly contributes to the positive VL shift. After irradiation, cases 1, 2, and 2’ show further negative VL shift due to the fact that the Al-Cl dipole is compressed more by the radiation pressure on the Pc plane. However, the VL shift for the case 3 appears positively large up to 0.38 eV. This implies the switch to Cl-up configuration, which will be explained later on. When the temperature of the samples is increased to RT, the cases 1, 2, and 2’ show similar positive VL shift by about 0.14 eV because of the bond-length increase of Al-Cl dipole and hence the strengthening of the interfacial Cl-Ag dipole. Specially for the cases 1 and 2, the contribution to the positive VL shifts also includes that from the tilting of Cl-down ClAlPc driven by the radiation pressure^12^. Post annealing to 60 °C drives case1 and case 2 to transit to the incomplete Cl-up configuration; both cases show similar increase in VL shift due to the existence of tilted configuration as indicated by the graphic illustration in the annotation for case1 and case 2. Yet the same treatment causes case 2’ to transform to the complete Cl-up configuration with negative VL shift as demonstrated in previous studies^12,21^ due to the pushback effect. For case 3, the complete Cl-up configuration already formed at the after-irradiation stage, as implied by the almost statured large VL shift about 0.4 eV throughout the last three stages. The large difference in VL shift between case 2’ and case 3 in spite of the common completeness in Cl-up configuration can be explained by the proposed model in Fig. 4 that Cl-up configuration formed by optomechanical effect (case 3) still keeps the Pc plane buckled to some extent so that the push-back effect exerted by Pc plane on Ag(111) surface decreases to cause VL to shift back to a lot higher value than that of case 2’. Moreover, such Cl-up configuration with buckled Pc plane upheld by the lobes appears surprisingly robust even upon annealing to 60 °C.

Figure 6b shows the EDCs of the four cases at the final stage of post annealing to 60 °C. Indeed, EDCs of case 1 and case 2 resemble each other, consistent with their similarity in VL shift. Their lower intensity and broader line shape of Cl-up molecular-energy-state peak at − 8.8 eV compared with case 2’ and case 3 reveal the incompleteness of Cl-up configuration. For case 2’ and case 3, the former has a bit higher intensity for molecular-energy-state peaks at − 3.5 and − 8.8 eV (peaks (A) and (B) in Fig. 3a) than the latter. Upon a close examination on the partial DOS distribution in Fig. 3c, one can see that the contributions from inner ligand ring and outer phenylene groups to either peak (A) or (B) are compatible for the typical flat-on Cl-up configuration. For the special upheld Cl-up configuration, as represented by case 3, the charge-transfer channel from Ag(111) via inner ligand ring, as indicated by the orange arrows in Fig. 1b, should substantially reduce, so peaks (A) and (B) for case 3 are lower than those of the typical flat-on Cl-up configuration as represented by case 2’. A further confirmation can be seen from the inset in Fig. 6b where the HOMO peak at about − 1.25 eV is only obvious for case 2’; this peak is mainly contributed from inner ligand ring according to the partial DOS shown in Fig. 3c.

### DFT support for the proposed model

**Figure 7:**
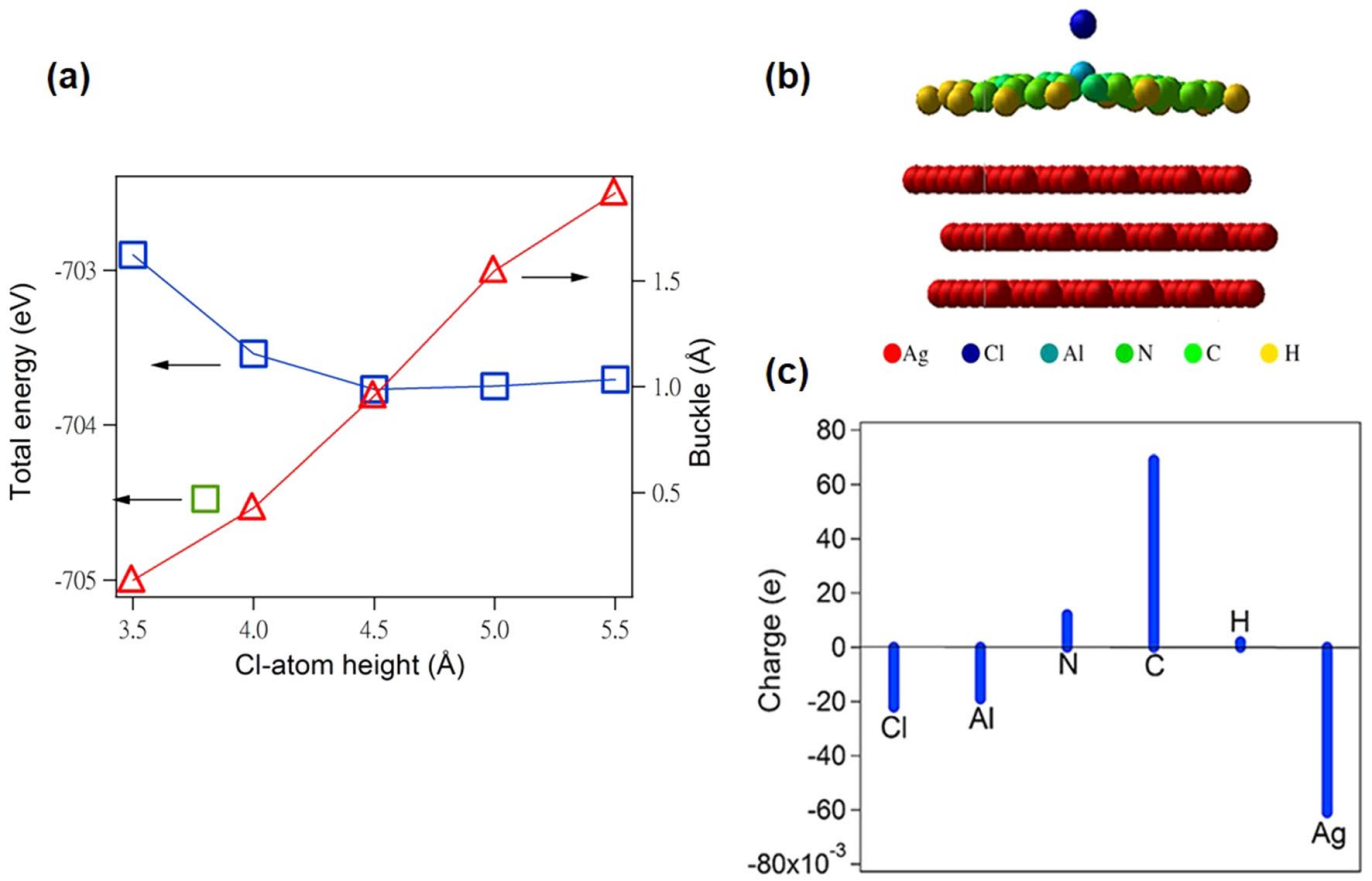
(**a**) Calculated total energy of ClAlPc/Ag(111) in Cl-up configuration as well as buckle values versus Cl-atom heights with respect to Ag(111) surface. (**b**) Side view of the Cl-up ClAlPc molecule with Cl-atom height 4.5 Å above Ag slabs. (**c**) The charge difference between the Cl-atom height at 4.5 Å and that at 3.5 Å over Cl, Al, N, C, H and Ag atoms.

The context for the proposed model in Fig. 4 based on the optomechanical effect is the robustness of two bent-down Pc lobes at the periphery of Cl-down ClAlPc, which strongly anchor at the Ag(111) surface. It sets up a robust structural frame through which Al-Cl dipole can be pushed by radiation pressure to tunnel through Pc plane to the Cl-up position, while keeping Pc lobes still bent down to Ag(111). The charge transfer from Ag(111) via the outer phenylene groups is thus crucial for the upheld Cl-up configuration. We employ DFT calculation to simulate upheld Cl-up configuration by fixing the Cl atom at several heights above the Ag(111) surface so that the charger transfer via inner ligand ring can be inhibited. Figure 7a shows the Cl-atom height dependence of the total energies as well as buckle values, i.e. the height difference between Al atom at the center and H atom at the periphery, derived from DFT calculation. As seen, there is an energy minimum corresponding to Cl-atom height of 4.5 Å with respect to Ag(111). Its side view is exhibited in Fig. 7b with the buckle value of 0.956 Å. Figure 7c shows the charge difference over individual atoms between the Cl-atom height at 4.5 Å and that at 3.5 Å with negligible buckle. It indicates the relevant charge transfer from the first Ag layer to C and N atoms for the former. In addition, the charges of Cl and Al atoms reduce for the former because of the negligible charge transfer through inner ligand ring. It has to be noted that our DFT result on the fully relaxed Cl-up ClAlPc on Ag(111) still points to the typical flat-on configuration as marked by the green square symbol at Cl-atom height of 3.8 Å with the total energy 0.7 eV lower than the energy minimum of the cases with fixed Cl-atom heights. In addition, our DFT result indicates the four Pc lobes of upheld Cl-up configuration all bend down toward Ag(111) surfaces. Upon the complete switchover from Cl-down to Cl-up configuration, it is likely that another two bent-up Pc lobes also bend down toward Ag(111) to strengthen stability so Cl-up ClAlPc evolves from C2 to C4 symmetry throughout the last two annealing stages while the Pc plane still maintains buckled. Such a situation likely leads to a compatible total-energy minimum to that of the typical flat-on Cl-up configuration. Nevertheless, this requires further detailed investigation by microscopic techniques such as STM.

## Conclusion

In conclusion, via experimental evidence extracted from measurements of time-dependent photoemission spectroscopy and VL shifts, we have demonstrated coherent optomechanical control of the ClAlPc polar-organic monolayer on a Ag(111) substrate, as it switches completely from the Cl-down to Cl-up configuration at low temperature of − 195 °C. Due to the Al-Cl bond contraction at low temperature and the relatively large cross-sectional area of the Pc plane, radiation pressure exerted on it effectively presses the Al-Cl dipole down toward the Ag(111) substrate (i) to strengthen the bond between the two Pc lobes and the Ag(111) consolidating their anchoring on the substrate, and (ii) induce tunneling of the Cl atom through the Pc plane to attain the switchover. Moreover, the Cl-up configuration formed this way is likely upheld by the Pc lobes. Thus, the interaction with the Ag(111) substrate is reduced, leading to an increased VL, that is ~ 0.4 eV higher than that of the typical flat-on Cl-up configuration. This provides a useful avenue toward work function tuning of metal electrodes in organic-based devices^33^. The optomechanical process referred to here is different from precedent studies, in which photoisomerization effect caused the switch between *cis* and *trans* states to induce mechanical motion. Our approach via irradiation pressure combined with the low-temperature condition is simple and straightforward, which can certainly be applied to a wide range of other functional polar organic molecules. Although STM has successfully proved the switchover between Cl-down and Cl-up configuration of a ClAlPc molecule via applying positive or negative bias, our work herein instigates a novel approach and opens a further possibility to coherently switch the configuration of an organic monolayer at microscopic level via radiation pressure. We expect that this approach shall become increasingly important with the rapid progress of modern optical technology.

## Supplementary Information

Supplementary Information.

## References

[1] Saywell A, Bakker A, Mielke J, Kumagai T, Wolf M, García-López V, Chiang PT, Tour JM, Grill L (2016). Light-induced translation of motorized molecules on a surface. ACS Nano..

[2] Suda M, Thathong Y, Promarak V, Kojima H, Nakamura M, Shiraogawa T, Ehara M, Yamamoto HM (2019). Light-driven molecular switch for reconfigurable spin filters. Nat. Commun..

[3] Li G, Song R, Ma W, Liu X, Li Y, Rao B, He G (2020). π-Extended Chalcogenoviologens with stable radical state enable enhanced visible-light-driven hydrogen evolution and static/dynamic electrochromic displays. J. Mater. Chem. A.

[4] Suda M, Takashina N, Namuangruk S, Kungwan N, Sakurai H, Yamamoto HM (2017). N-Type Superconductivity in an organic mott insulator induced by light-driven electron-doping. Adv. Mater..

[5] Hugel T, Holland NB, Cattani A, Moroder L, Seitz M, Gaub HE (2002). Single-molecule optomechanical cycle. Science.

[6] Comstock MJ, Levy N, Kirakosian A, Cho J, Lauterwasser F, Harvey JH, Strubbe DA, Fréchet JMJ, Trauner D, Louie SG, Crommie MF (2007). Reversible photomechanical switching of individual engineered molecules at a metallic surface. Phys. Rev. Lett..

[7] Comstock MJ, Strubbe DA, Berbil-Bautista L, Levy N, Cho J, Poulsen D, Fréchet JMJ, Louie SG, Crommie MF (2010). Determination of photoswitching dynamics through chiral mapping of single molecules using a scanning tunneling microscope. Phys. Rev. Lett..

[8] Ichimura K, Oh S-K, Nakagawa M (2000). Light-driven motion of liquids on a photoresponsive surface. Science.

[9] Zhu F, Tan S, Dhinakaran MK, Cheng J, Li H (2020). Light-driven macroscopic directional motion of water droplet on an azobenzene–calixarene modified surface. Chem. Commun..

[10] Quick M, Dobryakov AL, Gerecke M, Richter C, Berndt F, Ioffe IN, Granovsky AA, Mahrwald R, Ernsting NP, Kovalenko SA (2014). Photoisomerization dynamics and pathways of trans- and cis-azobenzene in solution from broadband femtosecond spectroscopies and calculations. J. Phys. Chem. B.

[11] Yang D, Piech M, Bell NS, Gust D, Vail S, Garcia AA, Schneider J, Park C-D, Hayes MA, Picraux ST (2007). Photon control of liquid motion on reversibly photoresponsive surfaces. Langmuir.

[12] Arumugam K, Chen HM, Dai JH, Gao MF, Goyal A, Lin MK, Nakayama Y, Pi TW, Metz S, Papadopoulos TA, Jeng HT, Tang SJ (2019). Using irradiation effect to study the disparate anchoring stabilities of polar-organic molecules adsorbed on bulk and thin-film metal surfaces. Appl. Surf. Sci..

[13] Li Huang Y, Lu Y, Niu TC, Huang H, Kera S, Ueno N, Wee ATS, Chen W (2012). Reversible single–molecule switching in an ordered monolayer molecular dipole array. Small.

[14] Liu SW, Lee CC, Yuan CH, Su WC, Lin SY, Chang WC, Huang BY, Lin CF, Lee YZ, Su TH, Chen KT (2015). Transparent organic up conversion devices for near-infrared sensing. Adv. Mater..

[15] Jiang Q, Xing Y (2020). Improved performance of small molecule organic solar cells by incorporation of a glancing angle deposited donor layer. Sci. Rep..

[16] Li L, Hu W, Fuchs H, Chi L (2011). Controlling molecular packing for charge transport in organic thin films. Adv. Energy Mater..

[17] Huang YL, Wang R, Niu TC, Kera S, Ueno N, Pflaum J, Wee ATS, Chen W (2010). One dimensional molecular dipole chain arrays on graphite via nanoscale phase separation. Chem. Comm..

[18] Huang YL, Chen W, Bussolotti F, Niu TC, Wee ATS, Ueno N, Kera S (2013). Impact of molecule-dipole orientation on energy level alignment at the submolecular scale. Phys. Rev. B.

[19] Zhang JL, Xu JL, Niu TC, Lu YH, Liu L, Chen W (2014). Reversible switching of a single-dipole molecule imbedded in two-dimensional hydrogen-bonded binary molecular networks. J. Phys. Chem. C..

[20] Lin MK, Nakayama Y, Zhuang YJ, Su KJ, Wang CY, Pi TW, Metz S, Papadopoulos TA, Chiang TC, Ishii H, Tang SJ (2017). Control of the dipole layer of polar organic molecules adsorbed on metal surfaces via different charge-transfer channels. Phys. Rev. B.

[21] Chiniwar S, Huang A, Chen TY, Lin CH, Hsing CR, Chen WC, Cheng CM, Jeng HT, Wei CM, Pai WW, Tang SJ (2019). Substrate-mediated umklapp scattering at the incommensurate interface of a monatomic alloy layer. Phys. Rev. B.

[22] Kresse G, Hafner J (1993). Ab initio molecular dynamics for open-shell transition metals. Phys. Rev. B.

[23] Kresse G, Furthmüller J (1996). Efficient iterative schemes for ab initio total-energy calculations using a plane-wave basis set. Phys. Rev. B.

[24] Kresse G, Furthmüller J (1996). Efficiency of ab-initio total energy calculations for metals and semiconductors using a plane-wave basis set. Comput. Mater. Sci..

[25] Perdew JP, Burke K, Ernzerhof M (1996). Generalized gradient approximation made simple. Phys. Rev. Lett.

[26] Kresse G, Joubert D (1999). From ultrasoft pseudopotentials to the projector augmented-wave method. Phys. Rev. B.

[27] Blöchl PE (1994). Projector augmented-wave method. Phys. Rev. B.

[28] Fukagawa H, Hosoumi S, Yamane H, Kera S, Ueno N (2011). Dielectric properties of polar-phthalocyanine monolayer systems with repulsive dipole interaction. Phys. Rev. B.

[29] Niu T, Zhou M, Zhang J, Feng Y, Chen W (2013). Dipole orientation dependent symmetry reduction of chloroaluminum phthalocyanine on Cu(111). J. Phys. Chem. C..

[30] Ternes M, Lutz C, Hirjibehedin C, Giessibl F, Heinrich A (2008). The force needed to move an atom on surface. Science.

[31] Pathak PD, Vasavada NG (1970). Thermal expansion of NaCI, KCI and CsBr by X-ray diffraction and the law of corresponding states. Acta Cryst..

[32] Kuru Y, Wohlschlögel M, Welzel U, Mittemeijer EJ (2008). Coefficients of thermal expansion of thin metal films investigated by non-ambient X-ray diffraction stress analysis. Surf. Coat. Technol..

[33] Mandal S, Mukherjee M, Hazra S (2020). Evolution of electronic structures of polar phthalocyanine-substrate interface ACS. Appl Mater. Interfaces.

