# Supplementary material for: Optomechanical switching of adsorption configurations of polar organic molecules by UV radiation pressure
Source: Sci Rep. 2021 Jun 16;11:12645. doi: 10.1038/s41598-021-92046-w (PMC8209108; doi:10.1038/s41598-021-92046-w)
Supplement: Supplementary file 1 — Supplementary Information. [file 41598_2021_92046_MOESM1_ESM.docx]

Supplementary Information

**Optomechanical Switching of Adsorption Configurations of Polar Organic Molecules by UV Radiation Pressure**

Kowsalya Arumugam,^1^ Abhishake Goyal,^1^ Hong-Ming Chen,^1^ Jing-Huan Dai,^1^ Mau-Fu Gao,^1^ , Yasuo Nakayama,^2^ Tun-Wen Pi,^3^ Theodoros A. Papadopoulos,^4*^ Horng-Tay Jeng,^1,5,6ξ^ and Shu-Jung Tang^1,3, 6#^

1. *Department of Physics and Astronomy, National Tsing Hua University, Hsinchu 30013, Taiwan, ROC*

2*. Department of Pure and Applied Chemistry, Tokyo University of Science, 2641 Yamazaki, Noda, Chiba 278-8510, Japan*

3. *National Synchrotron Radiation Research Center (NSRRC), Hsinchu 30076, Taiwan, ROC*

4. *Department of Mathematical and Physical Sciences, University of Chester, Thornton Science Park, Chester CH2 4NU, U.K.*

5*. Physics Division, National Center for Theoretical Sciences, Hsinchu 30013, Taiwan, ROC*

6*. Institute of Physics, Academia Sinica, Taipei 11529, Taiwan, ROC*

The schematic model (Figure 3(d) of Ref. 12) to illustrate the flipping process from Cl-down to Cl-up configuration under irradiation impinging at room temperature. Note the broad spectral line shape of Cl-up molecular energy state in the photoemission spectrum indicates tilted and Cl-up configurations coexist after irradiation as shown from Figure 4(b) of Ref. 12.

T = Room Temperature
